# Phenolic Composition and Skin-related Properties of the Aerial Parts Extract of Different *Hemerocallis* Cultivars

**DOI:** 10.3390/antiox9080690

**Published:** 2020-08-02

**Authors:** Katarzyna Szewczyk, Małgorzata Miazga-Karska, Wioleta Pietrzak, Łukasz Komsta, Barbara Krzemińska, Anna Grzywa-Celińska

**Affiliations:** 1 Department of Pharmaceutical Botany, Medical University of Lublin, 1 Chodźki Str., 20-093 Lublin, Poland; wioleta.pietrzak@umlub.pl (W.P.); barbara.krzem@gmail.com (B.K.); 2 Department of Biochemistry and Biotechnology, Medical University of Lublin, 1 Chodźki Str., 20-093 Lublin, Poland; malgorzata.miazga-karska@umlub.pl; 3 Department of Medicinal Chemistry, Medical University of Lublin, 4 Jaczewskiego, 20-090 Lublin, Poland; lukasz.komsta@umlub.pl; 4 Chair and Department of Pneumonology, Oncology and Allergology, Medical University of Lublin, 8 Jaczewskiego Str., 20-090 Lublin, Poland; acelin@op.pl

**Keywords:** *Hemerocallis*, LC-ESI-MS/MS, polyphenols, antioxidant, antimicrobial, skin-aging

## Abstract

*Hemerocallis* plants are important vegetables with nutritional and health value, especially in eastern Asia, where they have been used as medicines to cure disease such as depression and inflammation for thousands of years. The present study concerns the determination of flavonoids and phenolic acids, as well as antioxidant, anti-collagenase, anti-elastase, anti-tyrosinase and antimicrobial properties of taxa cultivated in Poland. For chemical composition estimation, LC-ESI-MS/MS analysis and spectrophotometric assays were performed. The results show the presence of sixteen compounds in all analyzed species. Among the investigated cultivars, it was found that *H.* “Chicago Apache” and *H. fulva* var. *kwanso* have the highest total phenolic acid and flavonoid content. The most abundant compounds in all analyzed extracts were chlorogenic acid (209.8 to 1010.0 µg/g of DE) and quercetin-3-*O*-rutinoside (114.7 to 1049.7 µg/g of DE). The studied extracts exhibited moderate to high skin-related activities. These properties were correlated with a high concentration of polyphenols. The present study demonstrated that *Hemerocallis* cultivars contain significant amounts of phenolic compounds with good skin-related activities and could be interesting as novel sources of bioactive agents for the pharmaceutical, food and cosmetic industries.

## 1. Introduction

Plant extracts are a valued source of biologically active compounds. Numerous scientific studies confirm the antibacterial, antiviral, antifungal and anti-inflammatory effects of plant raw extracts [1]. Cosmetic products containing them have been gaining popularity in recent years. Their use in cosmetic preparations yield much better results than the application of single biologically active compounds. Using the health-promoting properties of plant substances, one primarily looks for ingredients with anti-inflammatory and antioxidant effects (that eliminate adverse changes in the body), as well as with a positive impact on the functioning of the skin. An example of such case is materials rich in polyphenols, such as phenolic acids and flavonoids, as natural antioxidants efficient in preventing free radical production. Phenolic acids, which have a protective role in many illnesses such as cancer, inflammation, and cardiovascular illnesses, particularly recognized due to their potent antioxidant capacity, are the primary polyphenols produced by plants and are used in the health care, food and cosmetics industries [2]. There is a lot of evidence showing a strong correlation between phenolic content and antioxidant potential [3,4]. Moreover, polyphenols, due to their ROS-scavenging activity and capacity to chelate metals, possess a pigment-reducing action and are often used in skin-lightening formulas [5]. Therefore, plant materials are often used in the cosmetics industry, mainly in anti-aging products. Currently, several edible and ornamental plants, especially used in folk medicine, can be more and more often found in modern cosmetic preparations [1,5].

The *Hemerocallis* (daylily) genus belongs to the *Asphodelaceae* family and *Hemerocallidoideae* subfamily [6]. These taxa are mainly cultivated as ornamental plants in China and American and European countries [7,8]. However, daylilies also possess nutritional and health value [8,9], and are used, especially in Asian traditional medicine, as antiemetic, anthelmintic, antispasmodic, antiphlogistic, diuretic and sedative remedies [10,11,12,13]. The ethnic groups of the Lohit District of Arunach Pradesh in India traditionally use rhizome paste of *H. fulva* (local name—Kuankai) in the cure of inflammation-related diseases such as fire burn skin [14]. Some daylily species are also used in East-Asian folk medicine against depression [15]. Moreover, flowers of different *Hemerocallis* species have been used in eastern Asia as functional and regular food [16]. Literature studies have shown that *Hemerocallis* contains steroidal saponins [17], polyphenols [11,16,18,19], antraquinones [10], carotenoids [20], naphthalene glycosides [7], essential oils [21,22] and lactams [23,24]. Extracts from the aerial parts and roots have numerous activities, such as anticancer [25], anti-inflammatory [26,27], antidepressant [28,29], antioxidant [7,22,30,31,32,33], and neurological activities [34]. Moreover, many patents have shown that extracts prepared from these species have potential use in cosmetics, e.g., as whitening ingredients [35,36,37,38].

As a response to the growing demand for high-quality cosmetics, one can see an increasing interest in products containing natural plant extracts that are rich in many active substances. In this study, the authors attempted to demonstrate benefits arising from the potential use of edible and ornamental plants, such as *Hemerocallis* cultivars. As part of the effort to discover new functional components for anti-aging and skin-whitening preparations, eight *Hemerocallis* cultivars cultivated in Poland were investigated, estimating their antioxidant, anti-collagenase, anti-elastase, anti-tyrosinase and antimicrobial properties as well as flavonoid and phenolic acid content.

## 2. Materials and Methods

### 2.1. Chemicals and Reagents

Ascorbic acid, collagenase from *Clostridium histolyticum*, 2,2-diphenyl-1-picrylhydrazyl radical (DPPH), elastase from porcine pancreas, (-)-epigallocatechin gallate (EGCG), Folin–Ciocalteu reagent, tyrosinase from mushroom (≥1000 unit/mg solid), ethylenediaminetetraacetic acid, disodium dihydrate (Na_2_EDTA*2H_2_O), Levodopa (L-DOPA), N-[3-(2-Furyl)acryloyl]-Leu-Gly-Pro-Ala (FALGPA), N-Succinyl-Ala-Ala-Ala-p-nitroanilide (SANA), Tricine (≥99%; titration) were obtained from Sigma-Aldrich (Steinheim, Germany). Phosphate-buffered saline (PBS) was purchased from Gibco (Carlsbad, CA, USA). Reference substances were from ChromaDex (Irvine, CA, USA). Acetonitrile, formic acid and water for LC analysis were from Merck (Darmstadt, Germany). All others chemicals were of analytical grade and were obtained from Polish Chemical Reagent Company (POCH, Gliwice, Poland).

### 2.2. Plant Materials

The flowering aerial parts of eight *Hemerocallis* cultivars [*H. fulva* (L.) L. var. *kwanso* Regel (year of introduction to cultivation-1967)—H1, *H.* “Aten” (1981)—H2, *H.* “Bożena” (2004)—H3, *H.* “Catherine Woodbuery” (1981)—H4, *H.* “Chicago Apache” (2008)—H5, *H.* “Danuta” (2004)—H6, *H.* “Jaskółka” (2014)—H7, *H.* “Rebel Cause” (1983)—H8] were gathered in the Maria Curie-Skłodowska University (UMCS) Botanical Garden in Lublin (Poland), at altitude of 181.2 m a.s.l. (coordinates 51°15’46” N; 22°30’51” E) in August 2017. All details about these species, such as inventoty numbers and origin, were described in our previous research [22]. Taxonomic identification was confirmed by Dr. A. Dąbrowska, an employee of the Botanical Garden who specializes in *Hemerocallis*.

### 2.3. Preparation of the Extracts

The flowering aerial parts of *Hemerocallis* cultivars were dried at 40 °C (±2 °C) until they were at a constant weight. Powdered raw material (50 g) was macerated with mixture of ethanol and water (6:4, *v*/*v*) at an average temperature of 24.0 ± 0.5 °C for 24 h (3 × 150 mL). The combined extracts were filtered, concentrated under reduced pressure, then lyophilized in vacuum concentrator (Free Zone 1 apparatus; Labconco, Kansas City, KS, USA) to obtain dried residues.

### 2.4. Total Flavonoid, Phenolic and Phenolic Acids Content

Total flavonoid (TFC) and total phenolic content (TPC) were established using the colorimetric assays as described previously [39]. The absorbance was measured at 430 and 680 nm, respectively, using Pro 200F Elisa Reader (Tecan Group Ltd., Männedorf, Switzerland). Total phenolic concentration was estimated from the calibration curve (R^2^ = 0.9811), using gallic acid in (0.002–0.1 mg/mL) as a standard. The results were expressed as mg of gallic acid equivalent (GAE) per 1 g of dry extract (DE). Total flavonoid content was estimated from the calibrated curve (R^2^ = 0.9999), using quercetin (0.004–0.11 mg/mL) as a standard. The results were expressed as mg of quercetin equivalent (QE) per 1 g of DE. Total phenolic acids (TPAC) content was assayed using Arnov’s reagent as described in Polish Pharmacopoeia IX (an official translation of PhEur 7.0) [40]. The absorbance was measured at 490 nm. TPAC was estimated from the calibration curve (R^2^ = 0.9963), using caffeic acid in concentration 3.36–23.52 μg/mL as standard. The results were expressed as mg of caffeic acid equivalent (CAE) per 1 g of DE.

### 2.5. LC-ESI-MS/MS Analysis

Agilent 1200 Series HPLC system (Agilent Technologies, Palo Alto, CA, USA) coupled to 3200 QTRAP mass spectrometer (AB Sciex, Redwood City, CA, USA) was used for qualitative and quantitative analysis of flavonoids and phenolic acids in *Hemerocallis* extracts. The separation of analyzed compounds, injected in a 3-µL amount, was performed on a Zorbax SB-C_18_ analytical column (2.1 × 100 mm, 1.8 µm, Agilent Technologies, Palo Alto, CA, USA) at 25 °C. Elution was carried out using solvent A (0.1% HCOOH in water) and solvent B (0.1% HCOOH in acetonitrile). The following gradient elution program was used: 0–2 min—20% B, 3–4 min—25% B, 5–6 min—35% B, 5–6 min—35% B, 8–12 min—65% B, 14–16 min—80% B, 20–28 min—20% B. The flow rate was 300 µL/min. The mass spectra of analyzed compounds were acquired in the negative ESI mode, and the optimum values of the source parameters were as follows: capillary temperature 450 °C, nebulizer gas 50 psi, curtain gas 30 psi, source voltage −4500 V for phenolic acids and flavonoid glycosides, and capillary temperature 550 °C, nebulizer gas 30 psi, curtain gas 20 psi, and source voltage −4500 V for flavonoid aglycones analysis. Details of LC-ESI-MS/MS analysis are presented in Table 1 and were described in our previous research [41]. The Analyst 1.5 software (AB Sciex, Redwood City, CA, USA) was used for analysis and data acquisition. 

### 2.6. Antioxidant Activity 

All tests were made using 96. well plates (Nunclon, Nunc, Roskilde, Denmark) and were estimated in an Infinite Pro 200F Elisa Reader (Tecan Group Ltd., Männedorf, Switzerland). All experiments were performed in triplicate.

#### 2.6.1. DPPH Assay

2,2-diphenyl-1-picryl-hydrazyl (DPPH) free radical scavenging activity of *Hemerocallis* extracts and the ascorbic acid (positive control) was studied using a modified method, described previously [39]. Decreasing of DPPH absorbance, caused by the extracts, was controlled at 517 nm after incubation at 28 °C during 30 min. The results were expressed as IC_50_.

#### 2.6.2. Metal Chelating Activity (CHEL)

The metal chelating activity was established using method described by Guo et al., (2001) [3], modified in our previous study [39,42]. The absorbance was measured at 562 nm. As a positive control, Na_2_EDTA*2H_2_O was used.

Results were expressed as the IC_50_ values of the *Hemerocallis* extracts based on concentration–inhibition curves.

### 2.7. Enzyme Inhibitory Activity

All tests were made using 96-well plates (Nunclon, Nunc, Roskilde, Denmark) and were estimated in an Infinite Pro 200F Elisa Reader (Tecan Group Ltd., Männedorf, Switzerland). All experiments were performed in triplicate.

#### 2.7.1. Anti-Tyrosinase Activity (TYR)

Anti-tyrosinase activity was studied with the method described earlier by Zengin and co-authors [43]. Mushroom tyrosinase (40 µL, 200 U/mL) and *Hemerocallis* samples (25 µL) in different concentrations were incubated in sodium phosphate buffer (100 µL, pH 6.8) for 10 min at 29 °C. To start the reaction, L-DOPA (40 µL, 0.5 mM) was added. A blank sample was without a tyrosinase solution. The change in absorbance after 10 min incubation was measured at 492 nm at 29 °C. Kojic acid (6.25–100 µg/mL) was used as positive control. 

#### 2.7.2. Anti-Elastase Activity (ELA)

Anti-elastase activity was measured spectrophotometrically according to Chiocchio et al. [44]. Porcine pancreatic elastase (3.33 mg/mL; 25 µL) and *Hemerocallis* samples (in different concentrations) were incubated in Tris-buffer (0.2 mM, pH 8.0) for 10 min at 29 °C. To start the reaction, N-Succinyl-Ala-Ala-Ala-*p*-nitroanilide (2 mM; 125 µL) as a substrate was added. After 15 min incubation, the absorbance was measured at 420 nm. Epigallocatechin gallate (6.25–100 µg/mL) was used as positive control.

#### 2.7.3. Anti-Collagenase Activity (COL)

Anti-collagenase activity was studied using N-[3-(2-furyl) acryloyl]-Leu-Gly-Pro-Ala (FALGPA) as a substrate, and activity was measured according to Mandrone et al. [45]. Collagenase from *Clostridium histolyticum* (20 mU), dissolved in Tricine buffer (pH 7.5, 0.05 M, containing 0.4 M natrium chloride and 0.01 M calcium chloride), and *Hemerocallis* samples (in different concentrations) were incubated for 10 min at 29 °C. To start the reaction, FALGPA (1 mM) was added. After 15 min incubation at 29 °C, the absorbance was measured at 340 nm. Epigallocatechin gallate (6.25–100 µg/mL) was used as positive control. 

All results were expressed as the IC_50_ values of the *Hemerocallis* extracts based on concentration–inhibition curves.

### 2.8. Antibacterial Activity 

Zones of bacterial growth inhibition, caused by obtained extracts, were estimated for the reference microorganisms from the American Type Culture Collection (ATCC): Gram-positive bacteria (*Staphylococcus aureus* ATCC 25923) and Gram-negative bacteria (*Escherichia coli* ATCC 25992). Clinical strains (*Escherichia coli*, *Staphylococcus aureus*) isolated from infected wounds were obtained and stored since August 2016 in the strain bank at the Department of Biochemistry and Biotechnology, Medical University of Lublin, Lublin, Poland. Stock cultures were held at −70 °C. Tested bacterial strain, before the experiments, was passaged onto fresh Mueller–Hinton agar (M-H) (Oxoid, Basingstoke, UK) at 37 °C for 24 h. Next, inocula were made with fresh microbial cultures in sterile 0.9% NaCl to 0.5 McFarland turbidity standard. The antibacterial properties of extracts against bacteria was estimated by disk diffusion method, measuring the zones of inhibition. (Kirby-Bauer Disk Diffusion Susceptibility Test Protocol) on Petri plates with solid medium (M-H agar). Appropriate strain cultures were separately spread over the agar surface using a cotton swab. Next, tested liquid samples (100 μg) were placed using sterile disc (discs dispenser BioMaxima S.A., Lublin, Poland). After 18 h of incubation at 37 °C, zones of microbial growth produced around the studied extracts were estimated and recorded as the diameters of inhibition [mm]. All analyses were carried out in triplicate. The results are expressed as mean ± RSD.

### 2.9. Statistical Analysis

All results were stated as means ± standard deviation (SD) of three independent tests. One-way ANOVA with Tukey’s post hoc test was used for statistical analysis of significance of differences between means. *p* values below 0.05 were accepted as statistically significant. Statistics were carried out in Statistica 10.0, whereas Principal Component Analysis was carried out in R version 3.6.3 (64-bit, Windows 10), using built-in “prcomp” function.

## 3. Results and Discussion

### 3.1. Phytochemical Analysis

Total phenolic content (TPC) was determined using Folin–Ciocalteu reagent and the results were estimated as gallic acid equivalents (GAE) per g of dry extract (DE) (Table 2). Among eight *Hemerocallis* cultivars, *H.* “Chicago Apache” (H5) and *H. fulva* var. *kwanso* (H1) had the highest phenolic content (99.8 ± 1.1 mg GAE/g DE and 78.8 ± 0.4 mg GAE/g DE, respectively), followed by *H.* “Jaskółka” (H7) (45.5 ± 0.0 mg GAE/g DE), *H.* “Danuta” (H6) (35.3 ± 0.5 mg GAE/g DE), *H.* “Aten” (H2) (28.0 ± 0.1 mg GAE/g DE), *H.* “Rebel Cause” (H8) (22.1 ± 0.4 mg GAE/g DE), *H.* “Bożena” (H3) (16.1 ± 0.5 mg GAE/g DE), and *H.* “Catherine Woodbuery” (H4) (9.0 ± 0.4 mg GAE/g DE). The values obtained in our study are slightly higher than those of Lin and co-authors (2011) for various extracts of the flowers of *H. fulva* (from 25.3 ± 2.7 to 34.6 ± 2.7 mg GAE/g of extract) and lower than those of *H. fulva* leaves (25 ± 0.001 to 749 ± 0.004 mg chlorogenic acid equivalent/g) [46]. Mao et al. [31] found high amounts of phenolic compounds in leaves of *H. fulva*, ranging from 41.25 to 160.42 mg/g of dry extract. Fu and Mao [47] recorded a range from 44.68 to 59.22 mg of polyphenols per kg of dry weight for five *Hemerocallis* cultivars from China, and these values were lower than those obtained in our study. Moreover, Stefaniak and Grzeszczuk [48] determined total phenolic content for *Hemerocallis* × *hybrida* flowers on the level equal to 2.06 ± 0.02 mg GAE/g per fresh weight. 

The total flavonoid content of the flowering aerial parts of *Hemerocallis* cultivars was estimated by previously described colorimetric method [39] and was expressed as quercetin equivalents (QE) per g of dry extracts. The results presented in Table 1 show that among studied species, a significantly higher content of total flavonoids was observed for *H.* “Chicago Apache” (H5) and *H. fulva* var. *kwanso* (H1) (25.4 ± 0.0 and 25.0 ± 0.1 mg QE/g DE, respectively). The high content was also noted for *H.* “Jaskółka” (H7) (13.8 ± 0.2 mg QE/g DE) and *H.* “Danuta” (H6) (12.8 ± 0.1 mg QE/g DE). The data for the flowering aerial parts of *Hemerocallis* cultivars were higher than those obtained for *H. fulva* flowers (10.4 ± 2.3 to 19.5 ± 1.3 mg catechin equivalent/g of extract) [32].

The total phenolic acids content (TPAC) in the studied extracts are presented in Table 2. The amounts were in the range from 8.3 ± 0.1 to 18.9 ± 0.2 mg CAE/g DE. The highest TPAC content was observed in H2 (18.9 ± 0.2 mg CAE/g DE), followed by H5 (17.2 ± 0.1 mg CAE/g DE) and H1 (16.2 ± 0.1 mg CAE/g DE). The smallest content was found in H4 (8.3 ± 0.1 mg CAE/g DE).

**Figure 1:**
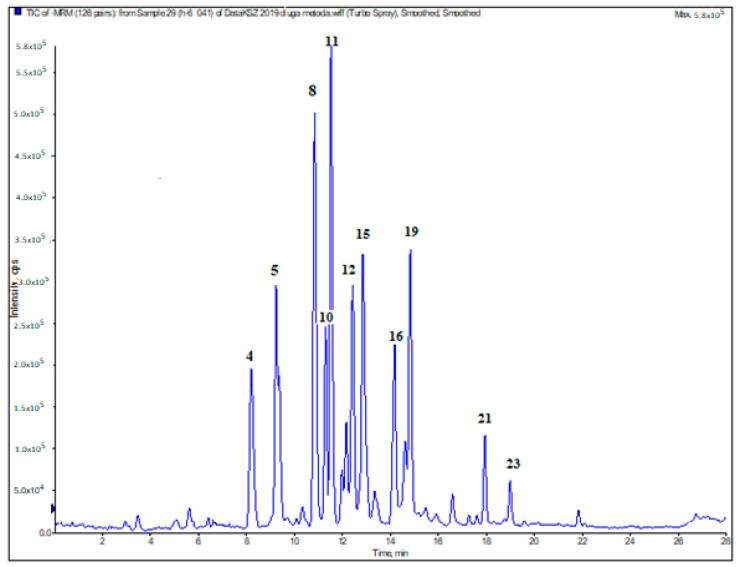
Representative LC-ESI-MS/MS total ion chromatogram of compounds presented in *H.* “Danuta” (H6). Peak numbers refer to those implemented in Table 3: 4—protocatechuic acid; 5—chlorogenic acid; 8—4-hydroxybenzoic acid; 10—*cis*-caffeic acid; 11—quercetin-3-*O*-rutinoside (rutin); 12—quercetin 3-*O*-galactoside (hyperoside); 15—isorhamnetin-3-*O*-rutinoside (narcissoside); 16—4-hydroxycinnamic acid (*p*-coumaric acid); 19—quercetin 3-*O*-rhamnoside (quercitrin); 21—quercetin; 23—isorhamnetin.

In the next step of our study, phenolic acid and flavonoid composition of the extracts obtained from *Hemerocallis* cultivars was investigated using the LC-MS/MS method. The analysis was carried out using a previously validated and described method [41]. The results of the qualitative and quantitative analysis are presented in Table 3. The sample LC-ESI-MS/MS chromatogram is displayed in Figure 1.

Among the investigated cultivars, it was found that *H.* “Chicago Apache” (H5) and *H. fulva* var. *kwanso* (H1) have the highest total phenolic acids and flavonoids content. Chlorogenic acid was the most abundant phenolic acid in all *Hemerocallis* extracts (209.8 ± 1.0 to 1010.0 ± 10.0 µg per g of DE). The protocatechuic, *p*-coumaric and caffeic acids were also observed in all *Hemerocallis* cultivars. The protocatechuic and caffeic acid contents of *H.* “Rebel Cause” (H8) extract (577.5 ± 12.5 and 98.0 ± 1.0 μg/g DE) were found to be much higher than those of the other extracts (45.9 ± 0.6 to 116.8 ± 0.5 μg/g DE for protocatechuic acid and from 10.0 ± 0.1 to 72.4 ± 1.9 μg/g DE for caffeic acid). As for *p*-coumaric acid, its content in the flowering aerial parts of *H.* “Aten” (H2) (53.1 ± 0.9 μg/g DE) was determined to be richer comparing to those of the other cultivars (11.8 ± 0.0 to 65.0 ± 0.8 μg/g DE). The 4-hydroxybenzoic acid was detected in a quantifiable amount only in H2–H6 extracts (5.1 ± 0.1 to 373.8 ± 1.3 μg/g DE) and rosmarinic acid only in H2 (11.9 ± 0.2 μg/g DE). 

The amounts of the flavonoid aglycones were the highest in the *H.* “Danuta” (H6) extract. These compounds were not found in *H.* “Aten” (H2), *H.* “Bożena” (H3) and *H.* “Catherine Woodbuery” (H4) extracts. The extract of *H.* “Danuta” (H6) (50.9 ± 1.1 μg/g DE) was higher than that of *H. fulva* var. *kwanso* (H1), *H.* “Chicago Apache” (H5), *H.* “Jaskółka” (H7) and *H.* “Rebel Cause” (H8) extracts in terms of quercetin (10.7 ± 0.1, 12.1 ± 0.1, 1.5 ± 0.1 and 42.3 ± 1.2 μg/g DE, respectively). Isorhamnetin was detected in quantifiable amount only in *H.* “Chicago Apache” (H5) and *H.* “Danuta” (H6) extracts (0.3 ± 0.0 and 6.0 ± 0.1 μg/g DE, respectively).

Among the obtained extracts, it was found that *H.* “Chicago Apache” (H5) and *H. fulva* var. *kwanso* (H1) have the highest total flavonoid glycoside content (3682.3 and 3067.2 μg/g DE, respectively). Narcissoside, rutin and quercitrin were determined in all cultivars studied. Quercetin-3-*O*-glucoside and kaempferol-3-*O*-rutinoside contents were the higher in *H. fulva* var. *kwanso* (H1) extract (1727.9 ± 13.9 and 183.4 ± 3.8 μg/g DE, respectively). The high amounts of these glycosides were also observed in *H.* “Chicago Apache” (H5) and *H.* “Jaskółka” (H7) samples. Isorhamnetin-3-*O*-glucoside was observed in a quantifiable amount only in H5 (245.1 ± 1.2 μg/g DE).

According to the literature reports, *Hemerocallis* species were found as the plants with high contents of phenolic acids and flavonoids. The chlorogenic acids in a methanolic extract of *Hemerocallis* were qualitatively profiled using LC–MS^3^. Three caffeoylquinic acids, three p-coumaroylquinic acids and two feruloylquinic acids were identified [49]. In studies of aqueous-methanol and methanol extracts of *Hemerocallis* cv. *Stella de Oro* flowers, isorhamnetin 3-*O*-glycosides, kaempferol and quercetin were isolated [7]. Moreover, fifteen phenolic compounds including, among others, kaempferol 3-*O*-{α-L-rhamnopyranosyl(1→6)[α-L-rhamnopyranosyl(1→2)]}-β-D-galactopyranoside, chrysoeriol 7-*O*-[β-D-glucuronopyranosyl(1→2)(2-*O*-*trans*-feruloyl)-β-D-glucuronopyranoside, chlorogenic acid methyl ester, chlorogenic acid, 4- and 5-caffeoylquinic acid, kaempferol, quercetin, astragalin, isoquercitrin, kaempferol 3-*O*-rutinoside, rutin, quercetin 3-*O*-{α-L-rhamnopyranosyl-(1→6)[α-L-rhamnopyranosyl(1→2)]}-β-D-galactopyranoside, were isolated from the aqueous-ethanolic extract of *H. fulva* flowers [32].

Therefore, our results are in agreement with previous investigations and it can be seen that extracts, especially aqueous-ethanolic, of *Hemerocallis* species are rich in terms of phenolics.

### 3.2. Skin-Related Activities

Skin aging is a natural, unavoidable, complex process, progressing over the years. Basic life functions disappear gradually, creating an incorrect response to external factors. Various intrinsic and extrinsic factors are responsible for this process, including hormonal, genetic, metabolic changes, as well as exposure to environmental stress [50]. As several enzymes and biomarkers are involved in the skin aging process, plant extracts, as inhibitors of specific enzymes involved in the aging, such as elastase or collagenase, are desirable. Thus, apart from qualitative and quantitative analysis of flavonoids and phenolic acids, this work focused on skin-related properties of *Hemerocallis* cultivars. In the study, we examined in vitro antioxidant, anti-collagenase, anti-elastase, anti-tyrosinase and antibacterial properties of eight *Hemerocallis* cultivars collected in Poland.

#### 3.2.1. DPPH Radical Scavenging Activity

The antioxidant activity was studied on the microplate scale in cell-free systems. All samples were studied in a concentration range from 1.25 to 40 mg/mL. The extracts of the flowering parts of *Hemerocallis* cultivars exhibited moderate to high scavenging capacity in a concentration-dependent manner. For comparison, the radical scavenging activity of ascorbic acid was tested in the same conditions. The higher DPPH scavenging activity was showed for the extract of *H.* “Chicago Apache” (H5) (IC_50_ = 0.9 ± 0.2 mg/mL) followed by *H. fulva* var. *kwanso* (H1) (IC_50_ = 1.1 ± 0.1 mg/mL). However, the IC_50_ value for *Hemerocallis* extracts was from two- to even forty-times higher (*H.* “Catherine Woodbuery”—IC_50_ = 19.4 ± 0.1 mg/mL) than the for ascorbic acid (IC_50_ = 0.5 ± 0.0 mg/mL) (Table 4).

The results of other published reports can be tough to compare due to the other conditions of experiments used. Nevertheless, several phenolic compounds isolated from *Hemerocallis* cv. *Stella de Oro* flowers were tested for their antioxidant activity. The flavonol 3-*O*-glycoside from this species demonstrated low antioxidant activities at 10 µM [7]. The scavenging effects of extracts of the flowers of six daylily cultivars from China have been tested on the DPPH radical [47]. The authors found that extracts of daylily cultivars in the concentration of 150 µg/mL possess the scavenging effect from 92.26 to 72.32%. These values are comparable to our findings.

#### 3.2.2. Metal Chelating Activity (CHEL)

It is well known that polyphenols can reduce oxidative stress by several mechanisms that depend on their chemical structure. One of these mechanisms is the chelation of metal ions, such as iron, which plays a key role in the production of harmful oxygen species [51]. Under normal conditions, iron is stored and transported by ferritin or transferrin, preventing the reaction of free iron ions with reactive oxygen species. The iron ions, by taking part in Fenton reaction, generate OH• radicals, which can react with lipids causing their peroxidation [52]. Therefore, it is important to search for new natural compounds with the potential ability to chelate metal ions. 

The chelating capacity was based on measuring the percentage of inhibition of ferrozine-Fe^2+^ complex formation. The extracts from *H. fulva* var. *kwanso* (H1), *H.* “Chicago Apache” (H5), *H.* “Danuta” (H6) and *H.* “Jaskółka” (H7) were the most active ones interfering with the formation of iron and ferrozine complexes, which suggests their high chelating capacity and ability to capture iron ions before ferrozine. These samples had IC_50_ values comparable (9.2 ± 0.1 µg/mL—H7 and 9.6 ± 0.1 µg/mL—H6) and higher (1.9 ± 0.3 µg/mL—H5 and 6.1 ± 0.6 µg/mL—H1) than Na_2_EDTA*2H_2_O (IC_50_ = 9.8 ± 0.0 µg/mL) used as positive control. Slightly lower activity was noted for *H.* “Bożena” (H3) with IC_50_ = 12.9 ± 0.1 µg/mL. The lowest chelating activity was found for extracts from *H.* “Catherine Woodbuery” (H4) and *H.* “Rebel Cause” (H8) (Table 4).

High levels of chlorogenic acid and rutin in the extracts of *Hemerocallis* cultivars may be responsible for their strong antioxidant effects [32], which was also confirmed in our study. 

#### 3.2.3. Anti-Collagenase Activity (COL)

Collagen is the predominant tissue constituent of normal human dermis which is mainly responsible for structural stability. The reduction of collagen is started by collagenases that split interstitial collagens [53]. The inhibition of collagenase activity can delay collagen degradation and thus delay wrinkle formation in aging skin. The anti-collagenase effect of eight *Hemerocallis* cultivars was measured using *C. histolyticum* collagenase. The results are presented in Table 5.

The extract of *H.* “Chicago Apache” (H5) showed the highest anti-collagenase activity with IC_50_ value of 38.5 ± 0.2 μg/mL. *H. fulva* var. *kwanso* (H1) and *H.* “Jaskółka” (H7) also showed high activity with IC_50_ values of 40.3 ± 0.4 μg/mL and 41.2 ± 0.3 μg/mL, respectively. These results were comparable to those achieved for EGCG, used as the positive control (IC_50_ = 34.9 ± 0.4 μg/mL). The lowest chelating activity was found for extracts from *H.* “Catherine Woodbuery” (H4) and *H.* “Rebel Cause” (H8) with IC_50_ = 83.8 ± 0.2 and 78.8 ± 0.4 μg/mL, respectively.

In many studies, it has been proved that polyphenols are mostly compounds that are responsible for collagenase inhibition [54,55]. Moreover, chlorogenic acid and quercetin derivatives, which were found in large quantities in all *Hemerocallis* extracts, have demonstrated a high collagenase inhibitory effect which was shown by different reports [54,56].

#### 3.2.4. Anti-Elastase Activity (ELA)

In normal adult skin, the elastin dominates, representing over 90% of the total content of a developed elastic fiber, and it is a protein responsible for the elasticity [53]. Because there are many reports showing that skin aging is directly related to the breakdown of elastin by the enzyme elastase [54], the elastase inhibitory activity was also determined for the daylilies extracts.

The analysis was achieved with using a N-Succinyl-Ala-Ala-Ala-p-nitroanilide (substrate molecule) and elastase from porcine pancreas (enzyme). The extracts were tested in various concentrations (6.25–100 μg/mL). EGCG was used as the positive control and it showed IC_50_ = 62.4 ± 0.1 μg/mL. As in the case of collagenase inhibition, the extract from *H.* “Chicago Apache” (H5) shows the highest anti-elastase activity with the IC_50_ value of 45.5 ± 0.1 μg/mL. The inhibition of elastase activity of *H. fulva* var. *kwanso* (H1) and *H.* “Jaskółka” (H7) was also high (IC_50_ = 51.0 ± 0.1 μg/mL and 56.7 ± 0.2 μg/mL, respectively). All the results of anti-elastase activity are presented in Table 5.

As with anti-collagenase activity, it has also been demonstrated that polyphenols are significant elastase inhibitors [57].

#### 3.2.5. Anti-Tyrosinase Activity (TYR)

Tyrosinase is a copper-containing enzyme widespread in nature, that is, a key enzyme in the biosynthesis of melanin, biopolymer responsible for the color of skin and hair, and also protect skin from ultraviolet [58]. Though melanin is a very important molecule, its overproduction in epidermal layers can cause various dermatological disorders, such as hyperpigmentation due to skin aging [54,58]. The hyperpigmentation in human skin, such as age spots, is not desirable, therefore it seems relevant to search for natural substances that inhibit the melanogenesis, especially tyrosinase inhibitors. 

In our study, the extracts of flowering aerial parts of *Hemerocallis* species and kojic acid used as positive control were tested in various concentrations (6.25–100 μg/mL). The extract from *H.* “Chicago Apache” (H5) was the most effective amongst all samples with IC_50_ = 16.6 ± 0.1 μg/mL. Other extracts showed slightly weaker activity with IC_50_ values from 20.6 ± 0.1 (H1) to 79.3 ± 0.2 μg/mL (Table 5).

Much research has been performed to identify the inhibitors of tyrosinase from natural sources [58,59] and it was found that flavonoids show high anti-tyrosinase activity [59]. It was noted that some flavonols, such as quercetin and kaempferol, can competitively inhibit tyrosinase activity by their ability to chelate metal, which leads to the irreversible inactivation of enzyme [58,59].

#### 3.2.6. Antibacterial Activity

The antibacterial activity of the *Hemerocallis* cultivars’ extracts against Gram-negative (*E. coli*) and Gram-positive (*S. aureus*, *S. epidermidis*) bacterial species was examined using a plate antimicrobial test. The obtained effects are presented in Table 6. All samples showed some activity against tested reference and clinical Gram-positive strains (zones in range of 4.4 ± 0.3 mm—27.3 ± 3.1 mm). The aerial parts of *Hemerocallis* “Chicago Apache” (H5) and *H.* “Jaskółka”(H7) showed the highest activity among the eight tested samples. The zones of reference *S. aureus* and *S. epidermidis* growth inhibition caused by *Hemerocallis* “Chicago Apache” (H5) were respectively 27.3 ± 3.1 mm and 19.5 ± 2.3 mm. Importantly, the growth of the tested clinical strains of *S. aureus* and *S. epidermidis* was also inhibited in highest amount by H5, creating zones of 19.5 ± 2.3 mm and 16.5 ± 2.1 mm, respectively. Inhibiting the growth of clinical strains is important when samples with potential therapeutic properties are tested. However, the tested samples showed no inhibition zones against Gram-negative *E. coli* strains. Therefore, *Hemerocallis* samples demonstrated narrow spectrum, limited only to Gram-positive bacteria, which may be beneficial in the prevention and fight against classic infections caused by Gram-positive strains.

The antibacterial effects of *Hemerocallis* cultivars are the first results published to date. As mentioned above, the strongest effects were found in extracts from *Hemerocallis* “Chicago Apache” (H5) and *H.* “Jaskółka”(H7). This may be ascribed possibly to an action of chlorogenic acid and rutin detected in high amounts in these extracts, which are known to cause damage to bacterial cell membranes, causing effluence of cellular matters, leading to the death of bacteria [60,61,62].

### 3.3. Multivariate Analysis of the Results

**Figure 2:**
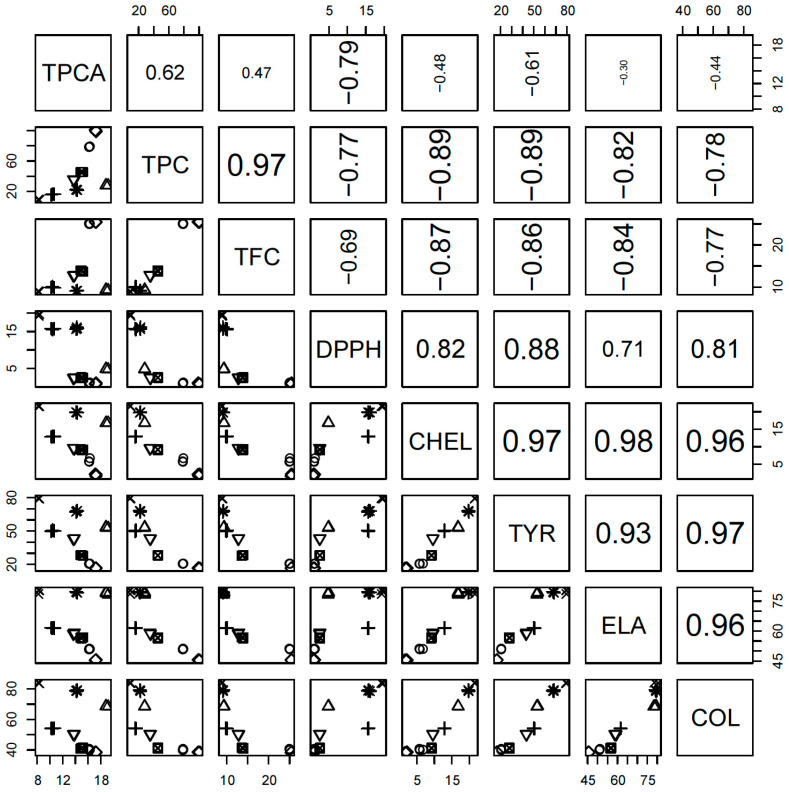
Pairwise plot of correlations between all investigated properties. TPCA—Total phenolic acids content; TPC—Total phenolic content; TFC—Total flavonoid content; CHEL—Metal chelating activity; TYR—Anti-tyrosinase activity; ELA—Anti-elastase activity; COL—Anti-collagenase activity. For explanation of species marks, see legend in Figure 4 caption

To perform a holistic view of the results, we used a chemometric multivariate approach. It allows us to examine some independent trends in changes among the investigated properties. During the introductory analysis of the correlations between investigated properties (Figure 2), one can conclude that DPPH, CHEL, TYR, ELA and COL are highly positively intercorrelated. On the contrary, they are negatively correlated with all other properties, mostly with TPC and TFC. TPCA is the last property, correlated positively with TPC and TFC and negatively with all other properties, however, these correlations are substantially lower. It should be underlined that negative correlations should be perceived and interpreted as positive in these cases, where the property is measured as the concentration with a particular effect (such as IC_50_). Then, the lower value denotes the higher activity. 

Therefore, we also performed scaled Principal Component Analysis of the whole data matrix, containing 24 rows (three samples for each eight species) and eight columns (all investigated properties). This approach allows us to examine intercorrelations between the properties, graph them in one two-dimensional graph and place the investigated samples in this reduced space. From a mathematical point of view, this is equivalent to the projection of multivariate space to the bivariate plane, where this plane is located in the best possible way (to visualize as much information, as possible). Of course, the visualized information depends on the data structure, so if the dataset is not intercorrelated (spherical), this method would not help us to understand the internal structure, as there is no optimal position on this plane.

The dataset was found to be easy to compress and 81.8% of the overall variance was located in the first PC. Of the variance, 11.6% was located in the second one, so the PC1-PC2 plot explains 93.3% of the overall variability inside this dataset. Therefore, almost all information can be presented in a two-dimensional projection and only several percentages of information are lost inside the orthogonal complement of this plane. It was also found that interesting information was located inside the PC3, explaining 5.1% of variance. No explainable trends were found in the further PCs.

**Figure 3:**
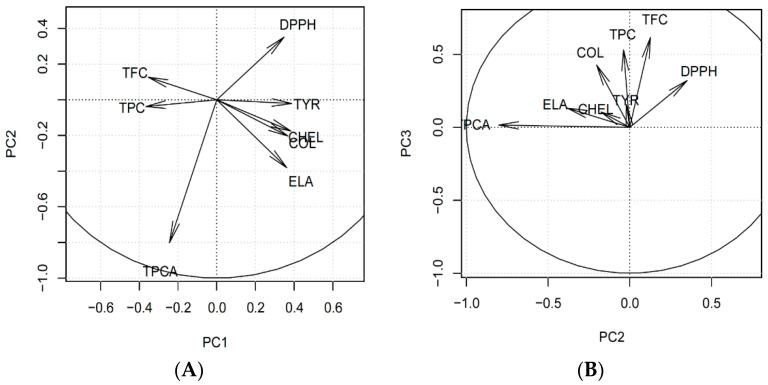
Principal Component Analysis loadings of the investigated dataset: (**A**) PC1 vs. PC2 plot, (**B**) PC2 vs. PC3 plot. TPCA—Total phenolic acids content; TPC—Total phenolic content; TFC—Total flavonoid content; CHEL—Metal chelating activity; TYR—Anti-tyrosinase activity; ELA—Anti-elastase activity; COL—Anti-collagenase activity.

The first main trend in this dataset, placed in PC1 (Figure 3) is connected (for high values of this PC) with simultaneous increase of DPPH, TYR, CHEL, COL and ELA, together with simultaneous decrease of TFC and TPC. Low PC1 values exhibit the opposite change. The highest PC1 was obtained for *H.* “Catherine Woodbuery” (H4) and *H.* “Rebel Cause” (H8), whereas the lowest for *H. fulva* var. *kwanso* (H1) and *H.* “Chicago Apache” (H5). It should be emphasized that species are spread uniformly according to the PC1 values, so this trend should be perceived as continuous and fluent. It can be concluded that flavonoids and phenols are responsible for all investigated biological activities, as the activities are expressed as IC_50_, and thus are negatively correlated with the content of particular compounds.

On the contrary, the second PC represents mainly the difference between *H.* “Aten” (H2), *H.* “Bożena” (H3), *H.* “Catherine Woodbuery” (H4) and *H.* “Rebel Cause” (H8) species. *H. fulva* var. *kwanso* (H1), *H.* “Chicago Apache” (H5), *H.* “Danuta” (H6) and *H.* "Jaskółka” (H7) have the second PC value close to zero. This trend is connected with increasing DPPH with a simultaneous decreasing of TPCA and slight decrease of ELA (H3, H4, high PC2) or opposite change (H2, H8, low PC2). This trend can be interpreted as antioxidant activity, orthogonal to all other activities and negatively correlated with TPCA. As DPPH is measured as the concentration, the dependence is opposite, and it proves that TPCA is mainly responsible for antioxidant activity.

**Figure 4:**
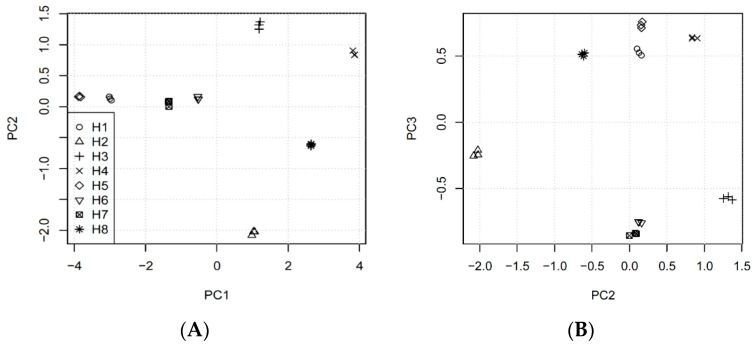
Principal Component Analysis scores of the investigated dataset: (**A**) PC1 vs. PC2 plot, (**B**) PC2 vs. PC3 plot. H1—*H. fulva* (L.) L. var. *kwanso* Regel, H2—*H.* “Aten”, H3—*H.* “Bożena”, H4—*H.* “Catherine Woodbuery”, H5—*H.* “Chicago Apache”, H6—*H.* “Danuta”, H7—*H.* “Jaskółka”, H8—*H.* “Rebel Cause”.

The third PC (Figure 4) presents the difference between H1, H4, H5 and H8 (high PC3) and the other species (low PC3). Species with high PC3 increased COL, TPC, TFC and (slightly) DPPH together (simultaneously) compared to the other species.

## 4. Conclusions

Due to the fact that natural ingredients in cosmetics are no longer just a trend, but a standard in creating new care products, there is a constant search for new plant raw materials. Plant extracts could contain specific secondary metabolites that provide a very wide range of possibilities in use.

The results of this study suggest that the *Hemerocallis* cultivars may have a value in the production of cosmetics. We found that aqueous-ethanol extracts of the flowering aerial parts of eight daylilies were rich in phenolics. Among the investigated cultivars, it was found that *H.* “Chicago Apache” and *H. fulva* var. *kwanso* have the highest total phenolic acids and flavonoids content. The most abundant compounds in all analyzed extracts were chlorogenic acid and quercetin-3-*O*-rutinoside. The studied extracts exhibited moderate to high skin-related activities. These properties were correlated with a high concentration of polyphenols. The present study demonstrated that *Hemerocallis* cultivars contain significant amounts of phenolic compounds with good skin-related activities, and could be interesting as novel sources of bioactive agents for the pharmaceutical, food and cosmetic industries.

## Figures and Tables

**Table 1:** Analytical results of LC-ESI-MS/MS quantitative method of phenolic acids and flavonoids. Limit of quantification (LOQ), limit of detection (LOD), and calibration curve parameters.

Compound	LOD [ng/mL]	LOQ [ng/mL]	R^2^	Linearity Range [ng/mL]
gallic acid	33.3	95.0	0.9987	167–3300
myricetin	5.0	10.0	0.9985	10–3600
kaempferol	20.0	33.0	0.9989	33–20,000
protocatechuic acid	17.0	34.0	0.9997	34–3470
chlorogenic acid	72.0	180.0	0.9991	180–18,000
*cis*-sinapic acid	17.4	69.4	0.9999	69.4–3470
rosmarinic acid	7.1	17.9	0.9994	17.9–7140
4-hydroxybenzoic acid	17.4	34.7	0.9993	69.4–3470
syringic acid	167.0	666.0	0.9993	666–11,100
*cis*-caffeic acid	60.0	160.0	0.9990	175–3500
gentisic acid	1.7	3.3	0.9997	3.3–330
vanillic acid	100.0	250.0	0.9997	330–33,000
caffeic acid	60.0	160.0	0.9990	175–3500
quercetin-3-*O*-rutinoside (rutin)	120.0	300.0	0.9985	2000–25,000
quercetin-3-*O*-galactoside (hyperoside)	150.0	200.0	0.9987	1000–25,000
quercetin-3-*O*-glucoside (isoquercetin)	150.0	300.0	0.9987	2000–25,000
kaempferol-3-*O*-rutinoside (nicotiflorin)	60.0	120.0	0.9991	120–50,000
isorhamnetin-3-*O*-rutinoside (narcissoside)	100.0	150.0	0.9985	200–2500
*p*-coumaric acid	7.3	18.1	0.9996	18.1–1820
kaempferol-3-*O*-glucoside (astragalin)	100.0	200.0	0.9978	1200–24,000
isorhamnetin-3-*O*-glucoside	100.0	250.0	0.9985	2000–20,000
quercetin 3-*O*-rhamnoside (quercitrin)	50.0	100.0	0.9986	1000–25,000
*o*-coumaric acid	7.3	18.1	0.9996	18.1–1820
quercetin	5.0	10.0	0.9980	20–3000
salicylic acid	3.3	16.5	0.9989	16.5–1650
isorhamnetin	15.0	30.0	0.9984	50–60,000

**Table 2:** The total phenolic (TPC), flavonoid (TFC) and phenolic acids (TPAC) content in the flowering aerial parts of *Hemerocallis* cultivars. H1—*H. fulva* (L.) L. var. *kwanso* Regel, H2—*H.* “Aten”, H3—*H.* “Bożena”, H4—*H.* “Catherine Woodbuery”, H5—*H.* “Chicago Apache”, H6—*H.* “Danuta”, H7—*H.* “Jaskółka”, H8—*H.* “Rebel Cause”; DE—Dry extract.

Sample	Total Phenolic Content [mg GAE/g DE]	Total phenolic Acids [mg CAE/g DE]	Total Flavonoid Content [mg QE/g DE]
H1	78.8 ± 0.4	16.2 ± 0.1	25.0 ± 0.1
H2	28.0 ± 0.1	18.9 ± 0.2	9.3 ± 0.1
H3	16.1 ± 0.5	10.4 ± 0.2	9.9 ± 0.1
H4	9.0 ± 0.4	8.3 ± 0.1	8.9 ± 0.1
H5	99.8 ± 1.1	17.2 ± 0.1	25.4 ± 0.0
H6	35.3 ± 0.5	13.8 ± 0.1	12.8 ± 0.1
H7	45.5 ± 0.0	15.0 ± 0.2	13.8 ± 0.2
H8	22.1 ± 0.4	14.2 ± 0.1	9.1 ± 0.0

**Table 3:** Content of phenolic acids and flavonoids in the flowering aerial parts of *Hemerocallis* cultivars. Mean values of three tests with standard deviation (± SD). Abbreviations: H1—*H. fulva* var. *kwanso*; H2—*H.* “Aten”; H3—*H.* “Bożena”; H4—*H.* “Catherine Woodbuery”; H5—*H.* “Chicago Apache”; H6—*H.* “Danuta”; H7—*H.* “Jaskółka”; H8—*H.* “Rebel Cause”; nd—Not detected; LOQ—Limit of quantification; DE—Dry extract; RT—Retention time; CE—Collision energy.

No	Compound	RT [min]	[M–H]^−^[*m*/*z*]	Fragment Ions [*m*/*z*]	CE [eV]	Amounts [µg/g DE]							
						H1	H2	H3	H4	H5	H6	H7	H8
1	gallic acid	5.07	168.7	78.9 124.9	−36 −14	<LOQ	<LOQ	nd	nd	<LOQ	<LOQ	nd	<LOQ
2	myricetin	5.95	316.7	136.9 150.9	−32 −26	nd	<LOQ	nd	nd	nd	<LOQ	nd	nd
3	kaempferol	7.56	284.7	116.8 93.0	−46 −52	nd	nd	nd	nd	<LOQ	<LOQ	nd	nd
4	protocatechuic acid	8.23	152.9	80.9 107.8	−26 −38	45.9 ± 0.6	74.5 ± 2.8	86.4 ± 1.1	63.2 ± 0.2	94.0 ± 2.0	116.8 ± 0.5	61.5 ± 1.5	577.5 ± 12.5
5	chlorogenic acid	9.30	352.9	190.8 84.9	−24 −60	982.5 ± 5.0	1010.0 ± 10.0	461.3 ± 11.3	356.3 ± 6.3	986.3 ± 8.8	209.8 ± 1.0	945.0 ± 5.0	291.3 ± 1.3
6	*cis*-sinapic acid	9.78	22.8	121.0 148.9	−36 −20	<LOQ	<LOQ	<LOQ	nd	nd	<LOQ	nd	nd
7	rosmarinic acid	10.23	358.7	132.6 160.8	−44 −20	nd	12.0 ± 0.2	nd	nd	nd	nd	nd	nd
8	4-hydroxybenzoic acid	11.27	136.8	92.9 107.9	−18 −18	<LOQ	20.6 ± 0.3	9.6 ± 0.2	46.8 ± 0.4	5.1 ± 0.1	373.8 ± 1.3	<LOQ	<LOQ
9	syringic acid	11.41	196.9	122.8 181.9	−24 −12	<LOQ	4.9 ± 0.2	<LOQ	<LOQ	<LOQ	<LOQ	<LOQ	<LOQ
10	*cis*-caffeic acid	11.68	178.7	88.9 134.9	−46 −16	45.4 ± 1.1	24.4 ± 0.9	13.3 ± 0.4	10.0 ± 0.1	21.2 ± 0.1	72.4 ± 1.9	10.8 ± 0.3	98.0 ± 1.0
11	quercetin-3-*O*-rutinoside (rutin)	11.99	608.7	299.6 270.9	−46 −60	927.9 ± 22.9	213.6 ± 5.3	251.6 ± 6.6	139.0 ± 2.5	1403.2 ± 43.7	114.7 ± 0.3	1049.7 ± 32.8	223.4 ± 1.9
12	quercetin-3-*O*-galactoside (hyperoside)	12.80	462.7	299.7 254.7	−28 −42	<LOQ	193.8 ± 2.2	214.7 ± 5.9	128.8 ± 3.9	<LOQ	319.6 ± 12.7	<LOQ	<LOQ
13	quercetin-3-*O*-glucoside (isoquercetin)	13.00	462.7	299.7 270.7	−30 −44	1727.9 ± 13.9	<LOQ	<LOQ	<LOQ	366.1 ± 2.1	<LOQ	1699.4 ± 51.6	238.8 ± 2.8
14	kaempferol-3-*O*-rutinoside (nicotiflorin)	13.31	592.7	284.8 226.7	−38 −68	183.4 ± 3.8	<LOQ	17.2 ± 0.4	127.2 ± 2.3	269.4 ± 9.6	<LOQ	105.5 ± 2.4	<LOQ
15	isorhamnetin-3-*O*-rutinoside (narcissoside)	13.52	622.8	314.9 298.8	−40 −52	151.4 ± 85.0	15.1 ± 0.1	29.7 ± 1.0	48.4 ± 0.6	1160.0 ± 19.5	49.7 ± 0.7	57.6 ± 2.1	<LOQ
16	*p*-coumaric acid	14.28	162.8	93.0 119.0	−44 −14	34.6 ± 0.4	53.1 ± 0.9	13.1 ± 0.2	11.8 ± 0.0	20.1 ± 0.6	65.0 ± 0.8	36.9 ± 1.2	19.1 ± 0.3
17	kaempferol-3-*O*-glucoside (astragalin)	14.66	446.7	226.8 254.8	−54 −40	<LOQ	<LOQ	<LOQ	<LOQ	<LOQ	nd	<LOQ	<LOQ
18	isorhamnetin-3-*O*-glucoside	14.76	476.8	313.9 270.9	−30 −44	<LOQ	<LOQ	<LOQ	<LOQ	245.1 ± 1.2	<LOQ	<LOQ	<LOQ
19	quercetin 3-*O*-rhamnoside (quercitrin)	14.83	446.7	299.7 270.7	−30 −40	76.6 ± 1.9	40.0 ± 0.3	51.3 ± 1.2	<LOQ	238.5 ± 7.7	376.3 ± 0.0	81.3 ± 1.2	123.9 ± 4.2
20	*o*-coumaric acid	17.17	162.8	119.0 93.0	−14 −46	<LOQ	nd	nd	nd	nd	nd	nd	nd
21	quercetin	17.91	300.7	150.9 178.8	−26 −20	10.7 ± 0.1	<LOQ	<LOQ	<LOQ	12.1 ± 0.1	50.9 ± 1.1	1.5± 0.1	42.3 ± 1.2
22	salicylic acid	18.06	136.9	75.0 93.0	−48 −16	<LOQ	<LOQ	<LOQ	<LOQ	<LOQ	<LOQ	<LOQ	<LOQ
23	isorhamnetin	19.09	314.7	299.7 150.9	−20 −30	<LOQ	<LOQ	<LOQ	<LOQ	0.3 ± 0.0	6.0 ± 0.1	<LOQ	<LOQ

**Table 4:** The IC_50_ values determined in antioxidant tests. Data are expressed as mean ± SD, *n* = 3. AA—ascorbic acid; Na_2_EDTA*2H_2_O—ethylenediaminetetraacetic acid, disodium dihydrate; nt—not tested; H1—*H. fulva* (L.) L. var. *kwanso*, H2—*H.* “Aten”, H3—*H.* “Bożena”, H4—*H.* “Catherine Woodbuery”, H5—*H.* “Chicago Apache”, H6—*H.* “Danuta”, H7—*H.* “Jaskółka”, H8—*H.* “Rebel Cause”; CHEL—metal chelating activity. * Statistically significant differences compared to AA; # statistically significant differences compared to Na_2_EDTA*2H_2_O, *p* < 0.05.

Sample	IC_50_	
	DPPH [mg/mL]	CHEL [μg/mL]
H1	1.1 ± 0.1 *	6.1 ± 0.6#
H2	4.8 ± 0.1 *	16.8 ± 0.0#
H3	15.7 ± 0.1 *	12.9 ± 0.1#
H4	19.4 ± 0.3 *	21.6 ± 0.0#
H5	0.9 ± 0.2	1.9 ± 0.3#
H6	2.5 ± 0.1 *	9.6 ± 0.1
H7	2.6 ± 0.0 *	9.2 ± 0.1#
H8	15.8 ± 0.3 *	19.2 ± 0.1#
AA	0.5 ± 0.0	nt
Na_2_EDTA*2H_2_O	nt	9.8 ± 0.0

**Table 5:** Anti-collagenase, anti-elastase and anti-tyrosinase activities of the flowering aerial parts of *Hemerocallis* cultivars. H1—*H. fulva* (L.) L. var. *kwanso* Regel, H2—*H.* “Aten”, H3—*H.* “Bożena”, H4—*H.* “Catherine Woodbuery”, H5—*H.* “Chicago Apache”, H6—*H.* “Danuta”, H7—*H.* “Jaskółka”, H8—*H.* “Rebel Cause”, nt—not tested. * Statistically significant differences compared to EGCG; # statistically significant differences compared to kojic acid, *p* < 0.05.

Sample	IC_50_ [µg/mL]		
	Collagenase Inhibition	Elastase Inhibition	Tyrosinase Inhibition
H1	40.3 ± 0.4 *	51.0 ± 0.2 *	20.6 ± 0.1#
H2	68.5 ± 0.2 *	78.9 ± 0.6 *	53.1 ± 0.4#
H3	54.1 ± 0.1 *	61.6 ± 0.1	50.2 ± 0.4#
H4	83.8 ± 0.2 *	79.8 ± 1.1 *	79.3 ± 0.2#
H5	38.5 ± 0.2 *	45.5 ± 0.1 *	16.6 ± 0.1#
H6	50.4 ± 0.2 *	59.0 ± 0.4 *	43.2 ± 0.2#
H7	41.2 ± 0.3 *	56.7 ± 0.2 *	28.0 ± 0.2#
H8	78.8 ± 0.4 *	79.4 ± 0.3 *	67.8 ± 0.4#
EGCG	34.9 ± 0.4	62.4 ± 0.1	nt
Kojic acid	nt	nt	17.6 ± 0.1

**Table 6:** The effect of the flowering aerial parts of *Hemerocallis* cultivars on the growth of Gram-positive and Gram-negative bacteria (mean ± RSD%, *n* = 3). H1—*H. fulva* (L.) L. var. *kwanso* Regel, H2—*H.* “Aten”, H3—*H.* “Bożena”, H4—*H.* “Catherine Woodbuery”, H5—*H.* “Chicago Apache”, H6—*H.* “Danuta”, H7—*H.* “Jaskółka”, H8—*H.* “Rebel Cause”.

Bacteria	Inhibition Zone Diameter (mm)							
	H1	H2	H3	H4	H5	H6	H7	H8
*Staphylococcus aureus* ATCC 25923	4.4 ± 0.3	8.0 ± 2.3	8.5 ± 1.9	14.0 ± 1.9	27.3 ± 3.1	11.8 ± 2.3	19.5 ± 2.1	9.9 ± 1.8
*Staphylococcus epidermidis ATCC 1222*	5.5 ± 2.2	4.0 ± 1.1	5.3± 2.2	9.5 ± 1.3	19.0 ± 0.1	14.3 ± 3.0	17.0 ± 0.4	11.7 ± 1.1
*Staphylococcus aureus* from wound	4.6 ± 1.4	5.5 ± 0.8	7.2 ±1.8	12.4 ± 3.5	19.5 ± 2.3	9.1 ± 1.0	14.0 ± 1.3	10.0 ± 0.9
*Staphylococcus epidermidis* from wound	4.7 ± 1.3	7.5 ± 1.2	6.1 ± 2.4	8.0 ± 0.9	16.5 ± 2.1	12.2 ± 1.6	15.0 ± 0.6	9.5 ± 1.8
*Escherichia coli ATCC* 25992	0	0	0	0	0	0	0	0
*Escherichia coli* from wound	0	0	0	0	0	0	0	0
